# TiN Films Deposited on Uranium by High Power Pulsed Magnetron Sputtering under Low Temperature

**DOI:** 10.3390/ma11081400

**Published:** 2018-08-10

**Authors:** Jingjing Ding, Xixi Yin, Liping Fang, Xiandong Meng, Anyi Yin

**Affiliations:** Institute of Material, China Academy of Engineering Physics, Mianyang 621900, China; dingjingjing21@126.com (J.D.); yinxixi@caep.com (X.Y.); fanglp26@163.com (L.F.); mengxiandong@caep.cn (X.M.)

**Keywords:** DU, TiN film, HPPMS

## Abstract

Depleted uranium (DU) is oxidized readily due to its chemical activities, which limits its applications in nuclear industry. TiN film has been applied widely due to its good mechanical properties and its excellent corrosion resistance. In this work, TiN protection films were deposited on DU by direct current magnetron sputtering (DCMS) and high power pulsed magnetron sputtering (HPPMS), respectively. The surface morphology and microstructures were investigated by atomic force microscope (AFM), scanning electron microscopy (SEM), and grazing incidence X-ray diffraction (GIXRD). The hardness and Young’s modulus were determined by nano-Indenter. The wear behavior and adhesion was analyzed by pin-on-disc tests and scratch adhesion tests and the corrosion resistance was evaluated by electrochemical measurements. The results show that the TiN films that were deposited by HPPMS outperformed TiN film deposited by DCMS, with improvements on surface roughness, mechanical properties, wear behavior, adhesion strength, and corrosion resistance, thanks to its much denser columnar grain growth structure and preferred orientation of (111) plane with the lowest strain energy. Besides, the process of Ti interlayer deposition by HPPMS can enhance the film properties to an extent as compared to DCMS, which is attributed to the enhanced ion bombardment during the HPPMS.

## 1. Introduction

Uranium is widely used in civilian and military applications due to its unique nuclear properties. However, it is chemically active and susceptible to corrosion, especially in salty, humid, and high-temperature environments [1,2]. Surface modification and film techniques have been applied to improve the corrosion resistance of U, including Ni [3], Al [4], Ti-based [5], Cr-based [6] films, and Mo^+^, C^+^, N^+^ [7,8] ion-implantation. These films can increase corrosion resistance to a certain extent. However, it is hard to get a pure surface without oxidation for films deposited on depleted uranium due to its high chemical activity. Inevitably, an undesirable interface adhesion and loose structure is induced. Consequently, the spallation failure of films and corrosion of the substrate easily occur. Thus, there exists an urgent demand for preparing a film with good performance to improve the serve life of depleted uranium (DU).

TiN films have been commonly used for protective purposes on different kinds of substrate materials due to its chemical stability and excellent mechanical properties [9,10,11,12,13]. However, the deposition temperature of TiN is above 300 °C in most reported literature [14], which is too high for DU for the following two facts: (1) the thick and loose uranium oxide layer that is formed at this temperature would decrease the interfacial adhesion strength between film and substrate intensively; (2) when uranium components are subjected to such high temperatures, they may deform and lose their mechanical properties. Until now, the film deposition under low temperature is still one of the most critical engineering and scientific problems that challenge the film application on uranium.

Efforts have been attempted to deposit TiN film on DU under low temperature by various methods including the conventional direct current magnetron sputtering (DCMS) and arc ion plating (AIP) techniques [5]. However, existing techniques encounter such difficulties as: the films deposited by DCMS at low temperature often exhibit a loose structure due to the low ionization rate of less than 1% [15]; AIP has high ionization rate, however, the macroscopic droplets produced in the film deposition process would result in some void defects and decrease the corrosion resistance consequently.

High power pulsed magnetron sputtering (HPPMS) is a novel technique in which the power provides the target with pulses of high power densities of a few kW cm^−2^. This technology enriched in metal ion plasma, could deposit dense structures and offer virtually defect free films at a relatively low temperature [16,17].

In this work, HPPMS technique was utilized to deposit dense TiN films on uranium for the first time. TiN films were deposited on DU by DCMS and HPPMS. The surface roughness, morphology, nano-hardness, adhesion strength, and electrochemical properties of these films were characterized in order to study the differences between conventional DCMS and HPPMS.

## 2. Materials and Methods

### 2.1. Sample Preparation

The Ti/TiN films were deposited on DU substrate and silicon (100) wafers, the later ones were prepared for phase and morphology analysis. The DU samples with a size of Ф15 mm × 3 mm were grinded using SiC water papers from 500# to 1000# progressively, mechanically polished to minor, and ultrasonically cleaned in acetone and ethanol, respectively, and then put into a vacuum chamber immediately when the substrates dried.

The base pressure was lower than 5 × 10^−4^ Pa and the chamber temperature was fixed to 180 °C. Prior to deposition, the DU samples were sputtered by Ar^+^ ions (2.0 Pa, applied bias voltage −800 V) for 30 min to clean the surface contaminations and partially remove the native oxide layer. After pre-sputtering, the Ti interlayer of approximately 100~300 nm thick was pre-deposited on the DU substrates to reduce the stress at the substrate interface and to enhance the adhesion between the film and the substrate. During deposition, the TiN films were deposited on the substrate with thickness of 4~5 μm.

Three deposition modes were designed to compare the structure and properties of the films deposited by HPPMS and DCMS, in order to investigate how the high power pulse introduced into magnetron sputtering process influences performance of the TiN films. Table 1 summarizes the deposition parameters for TiN film fabricated by DCMS, HP + DC, and HPPMS modes. In the DCMS mode, both Ti interlayer and TiN film were deposited by DCMS. In the HP + DC mode, the Ti interlayer was prepared by HPPMS, and then TiN layer by DCMS. However, in the HPPMS mode, both Ti interlayer layer and TiN film were deposited by HPPMS. The cathode was operated in a vacuum chamber equipped with a HPPMS power supply by employing the following parameters: frequency of 150 Hz, pulse width of 200 μs, and average power of 2.2 kW. The applied bias voltage of the substrate was −100 V. The deposition gas pressure was 0.3 Pa.

### 2.2. Film Characterization

The surface and cross-sectional morphology of the samples were observed by scanning electron microscopy (SEM, FEI 200, FEI, Hillsboro, OR, USA). The phase structure of the TiN films was characterized by grazing incidence X-ray diffraction (GIXRD, Philips X’Pert Pro, PANalytical B.V., Almelo, Netherlands) with Cu Kα radiation and the incident angle of 0.5°, where the scan range was from 30° to 80°. The Hardness and Young’s modulus of the deposited films were measured by a nano-Indenter (Triboindenter, Hysitron 950, Bruker, Billerica, MA, USA) with a Berkovich head. The test was applied with a load of 1 mN and depth of 90 nm. Then, the surface roughness of the TiN films was measured by an atomic force microscope (AFM, Hysitron, Bruker, Billerica, MA, USA) fitted to the indenter. The tribological test of the deposited films was performed on a ball-on-disc tribometer (Tribo-S-D, CSM, Peseux, Switzerland). In this test, SiN balls (diameter of 6 mm) were selected as friction pairs. The balls were under a constant normal load of 1 N, while the discs were circumrotated at a certain speed to generate abrasion of the films (detailed in Table 2). The scratch adhesion tests were performed on a Micro Combi Tester (Anton Parr, Graz, Austria), where the loading rate was 18 N/min and the progressive speed was 4 mm/min. Then, the scratch length was 3 mm. The tester was fitted with acoustic emission monitoring equipment, which can detect emission in the vicinity of 100 kHz. Scratches were examined by optical microscopy (OM, VHX-1000C, KEYENCE, Osaka, Japan) correlated with the acoustic emission observations to determine the cracking behavior and the critical loads (*L*c).

The corrosion behavior of DU and TiN coated DU samples were studied by potentiodynamic polarization techniques on an electrochemical workstation (PARSTAT2263, Ametek, San Diego, CA, USA). All of the electrochemical measurements were conducted using a conventional three-electrode electrochemical cell in aerated neutral NaCl solution with 50 μg/g Cl^−^ at room temperature with the specimen as working electrode, a platinum plate as counter electrode, and a saturated calomel electrode (SCE) as reference. The scan rate of potentiodynamic polarization was 0.2 mV/s.

## 3. Results and Discussion

### 3.1. Surface Morphology and Microstructure

**Figure 1:**
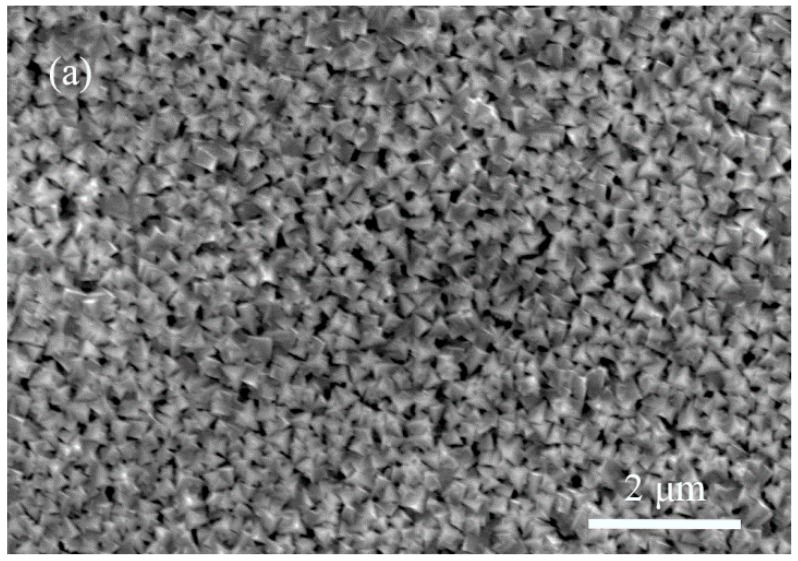

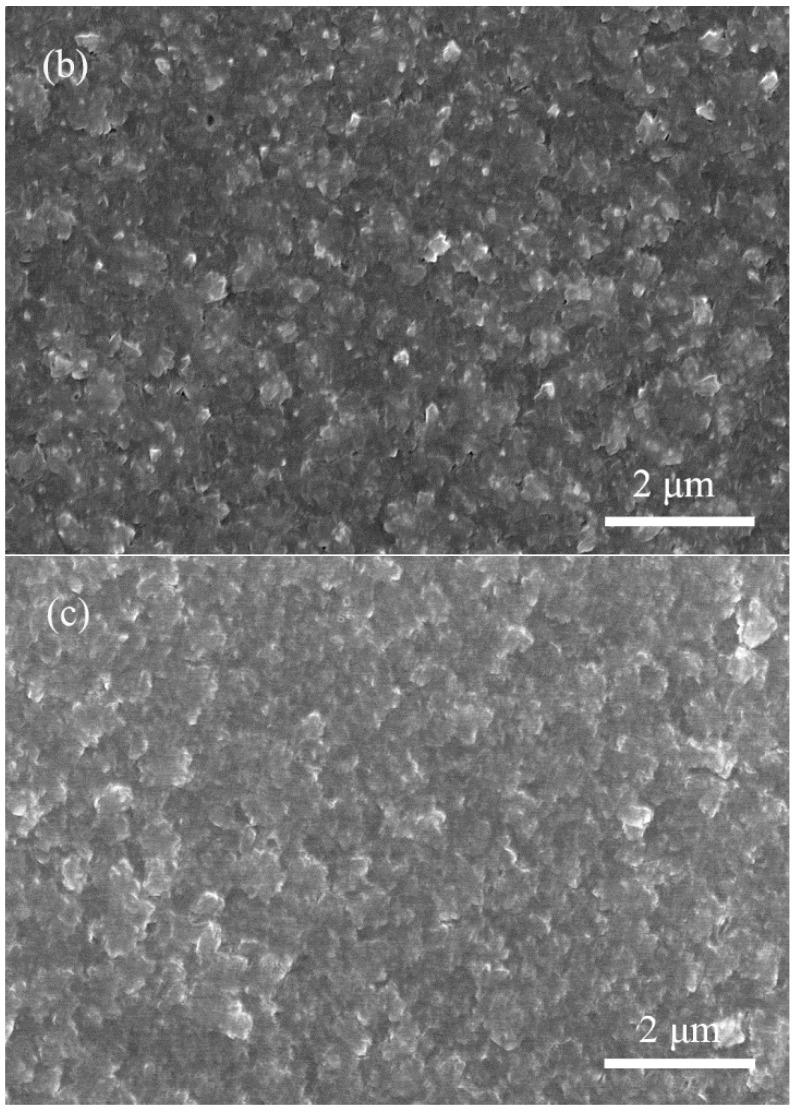
The surface morphology of TiN film by different deposition modes: (**a**) DCMS (**b**) HP + DC (**c**) HPPMS.

The surface morphologies of TiN film by different deposition modes are shown in Figure 1. The TiN film that was deposited by DCMS mode shows large micro particles with voids between each particle. While the film fabricated by HP + DC mode appears flattened and it interconnects with no presence of voids. In the case of HPPMS mode, the film presents the most compact and smoothest surface.

**Figure 2:**
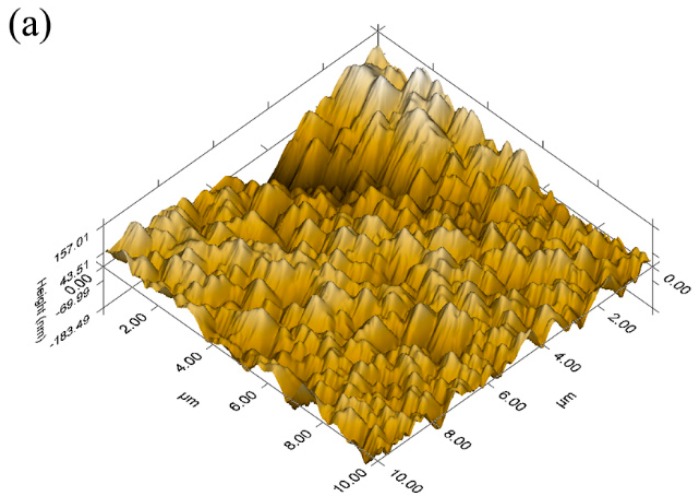

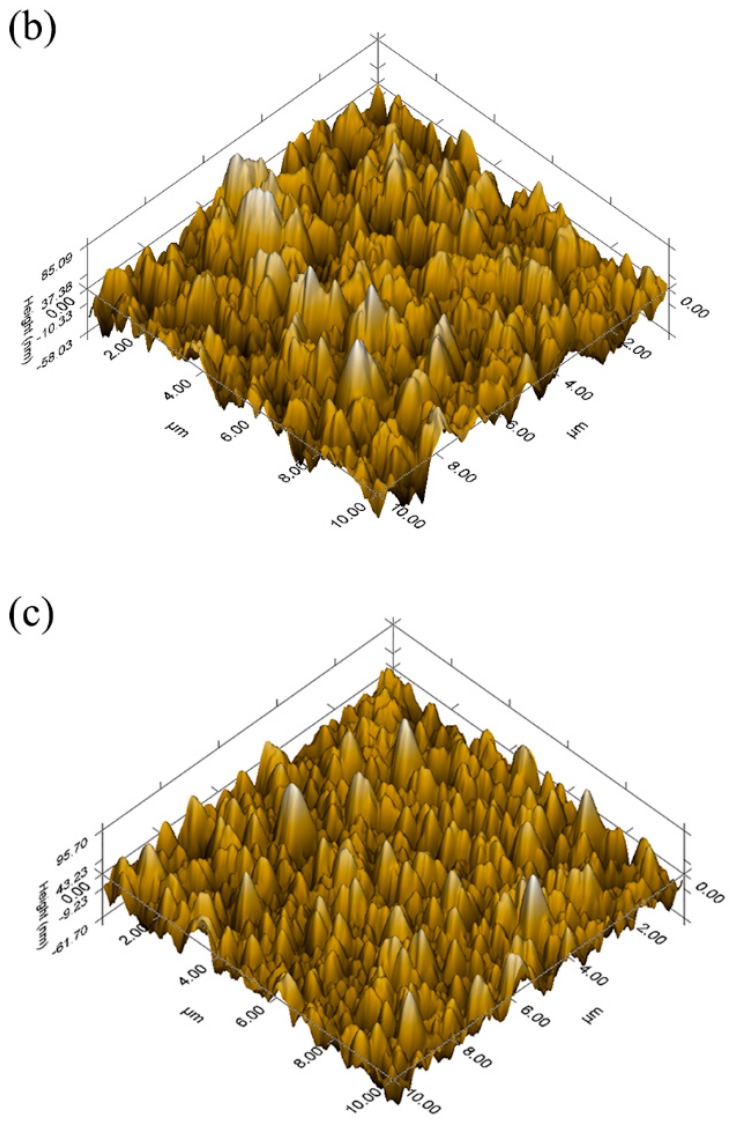
Atomic force microscope (AFM) images of TiN film by (**a**) DCMS; (**b**) HP + DC; and, (**c**) HPPMS mode.

Further, AFM was used to characterize the surface morphology of the deposited TiN films (Figure 2), and the averaged surface roughness (*R*_a_) of the samples was calculated accordingly (Table 3). The grain size of TiN decreased gradually from DCMS mode (~0.4 μm) to HPPMS mode (~0.3 μm). HPPMS mode will create a high ionization degree of target materials, which causes increased adatom energy and mobility on the substrate surface, and subsequently promotes the small grain migration to the grain boundaries and grain boundary migration to hence the refining grain sizes. The TiN film by DCMS mode has the roughest surface with *R*_a_ of 46.65 nm, while the film by the HPPMS mode presents the smoothest surface with *R*_a_ of 25.89 nm. This evolution can be attributed to the enhanced ion bombardment and subsequently higher surface energy of the growing films. Besides, the larger quantity of Ar^+^ ion bombardment at higher bias voltage also resulted in enhanced etching of the asperities and thus smoothing the surface.

**Figure 3:**
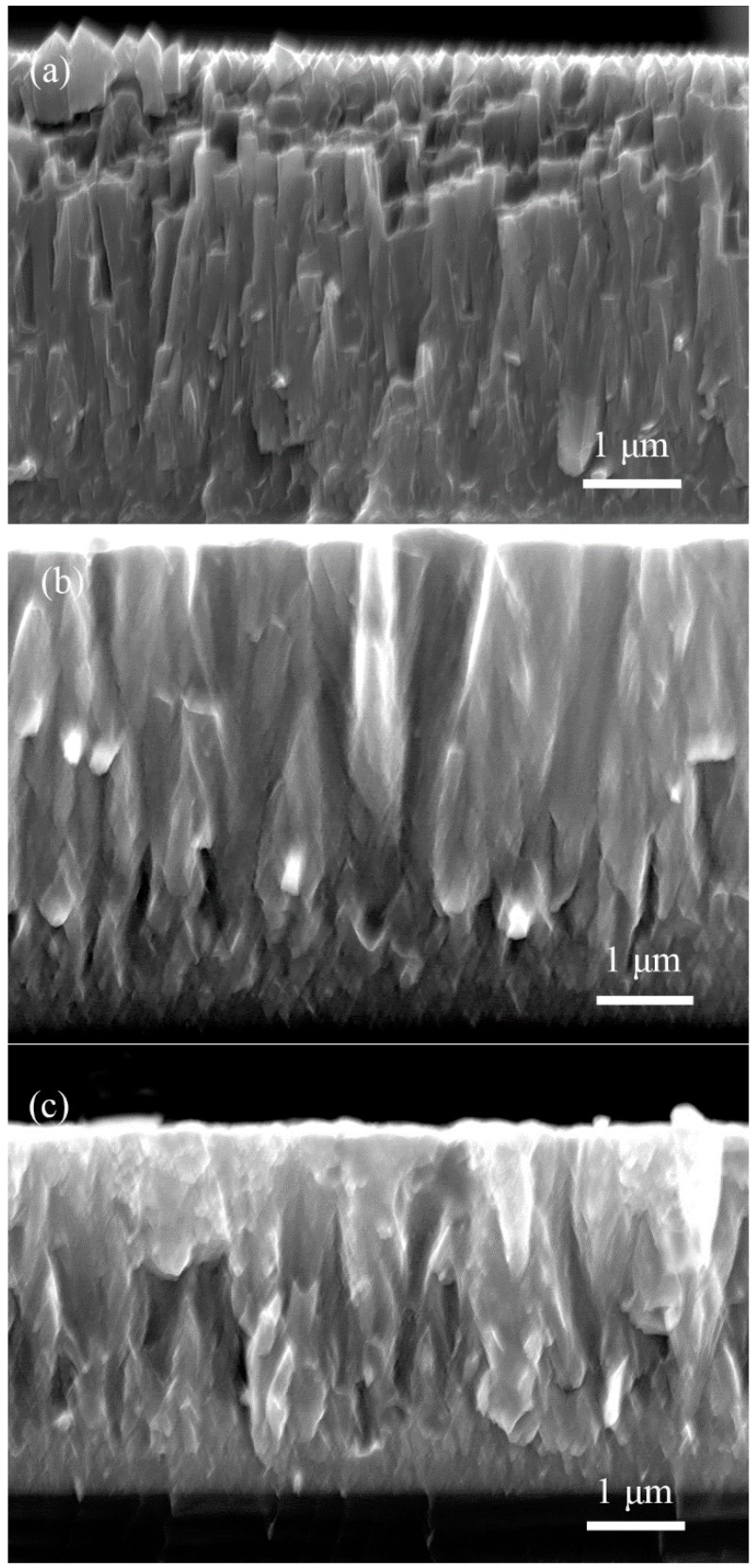
Cross-section morphology of TiN film grown by: (**a**) DCMS (4.7 μm); (**b**) HP + DC (4.7 μm); and, (**c**) HPPMS (4 μm).

**Figure 4:**
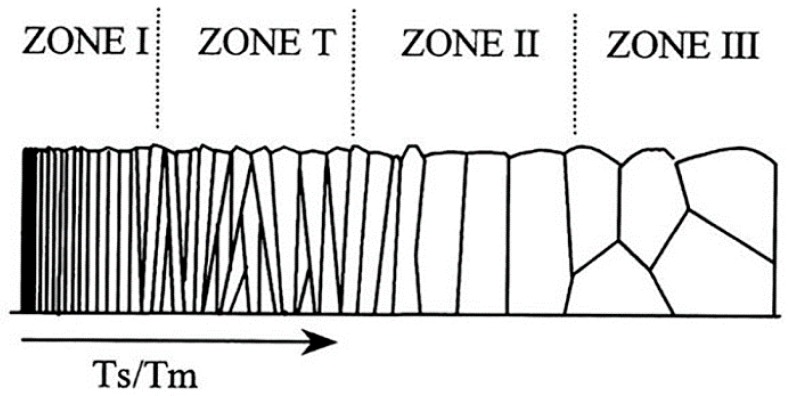
The structure zone model (SZM) [18].

Figure 3 presents the cross-sectional morphology of TiN film obtained by different deposition modes. The thickness of the as-deposited film was 4~4.7 μm. The film morphology was evaluated using the well-known structure zone models (SZM) (Figure 4) [18]. The film by DCMS mode presents a porous columnar morphology, which is the typical model of ZONE I. Due to the limited surface diffusion, the atoms that were deposited on the substrate failed to diffuse into the bulk. Hence, the film is consisted of uninterrupted fibrous columns, which exhibits porous and rough morphologies [19]. The HPPMS deposited film exhibits a rather denser columnar morphology identified by model of ZONE T. A high level energy of ion bombardment will enhance the surface diffusion, which gives rise to a different crystallographic orientation of grains, and therefore leads to a competitive growth. The structure mode of HP + DC deposited film is between the modes of ZONE I and T. The abundance of Ti^+^ ions in the Ti interlayer deposition flux during the HPPMS deposition will improve the transfer to the growth surface to a certain extent. However, in the subsequent DCMS mode where the majority of the ion flux consists of the much lower energy Ar^+^, the surface diffusion was limited.

**Figure 5:**
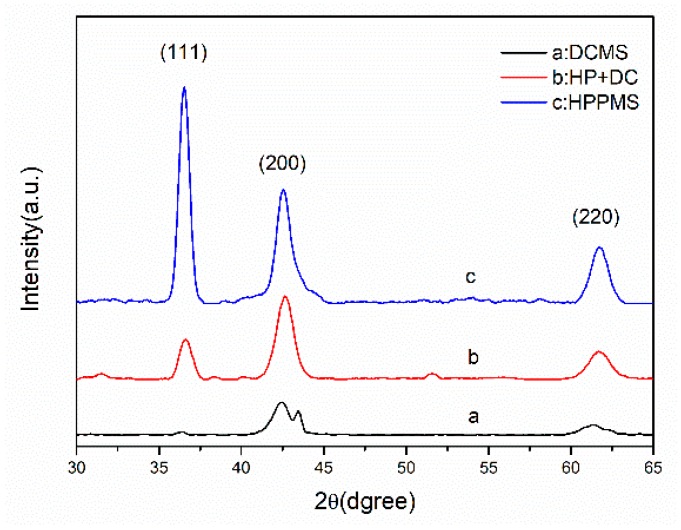
Grazing incidence X-ray diffraction (GIXRD) of TiN film deposited by three modes: (**a**) DCMS (**b**) HP + DC (**c**) HPPMS.

GIXRD measurements were performed on TiN films and the corresponding XRD patterns are shown in Figure 5. Figure 5a exhibits a strong (200) orientation, weak (220) orientation, and scarce (111) orientation. Figure 5b presents a mixture of more pronounced (200) orientation with less (111) and (220) orientation. Figure 5c exhibits (111) preferred orientation. It is proposed that the competition between surface energy and strain energy during film growth might contribute to the changes in preferred orientation [20]. The preferred orientation of film has an important effect on its performance [21]. As reported, the preferred orientation of (111) plane is the most closely packed and it exhibits the lowest strain energy, while (200) exhibits the lowest surface free energy [22,23]. In the DCMS mode, the applied substrate temperature is so low (*T*_s_ = 453 K) that the surface energy controlled the growth of the TiN film and the (200) preferred orientation favors [22]. In the case of HPPMS mode, the abundance of Ti^+^ ion bombardment increased the adatom mobility and diffusivity, thereby improving the strain energy, which facilitated the preferred orientation of (111) [23].

### 3.2. Hardness (H) and Young’s Modulus (E)

Hardness and Young’s modulus values of the film deposited by different modes are listed in Table 3. The hardness of HPPMS film (*H* = 20.56 GPa) was higher as compared to that of DCMS film (*H* = 15.75 GPa), and the hardness of HP + DC film (*H* = 22.09 GPa) was between them. The Young’s modulus exhibits a similar tendency. The enhanced hardness was attributed to both a dense microstructure without inter-columnar voids that were prepared during film growth in HPPMS discharge [24] and the fact that (111) is the hardest orientation in TiN [25].

### 3.3. Wear Behavior

**Figure 6:**
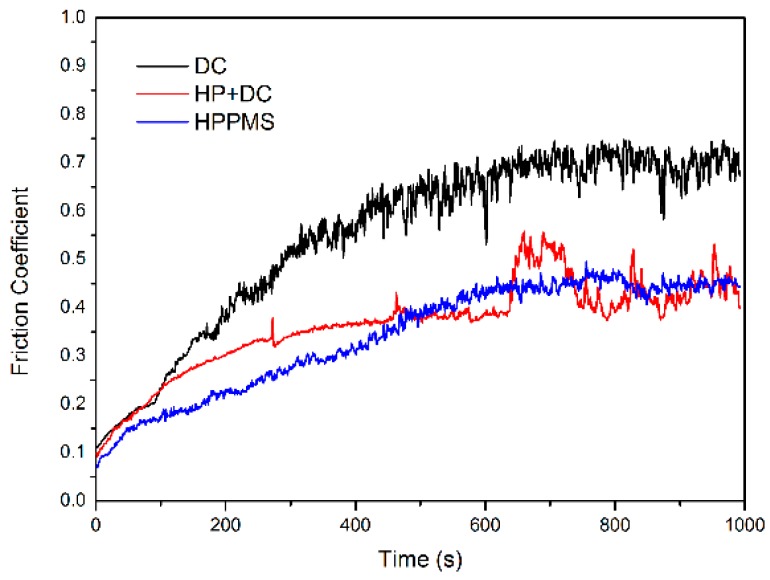
The film friction coefficient against sliding time by three modes.

The wear behavior of the TiN films was evaluated by pin-on-disc tests. Figure 6 plots the film friction coefficient against sliding time, and then Table 4 shows the corresponding average tribology coefficient (*μ*) of TiN film. It can be identified that the DCMS TiN has the highest wear coefficient (*μ* = 0.56). The film by HP + DC mode exhibits a reduced friction coefficient (*μ* = 0.42). While, the HPPMS deposited one has a further improved wear resistant (*μ* = 0.34) when compared to the two modes described above.

**Figure 7:**
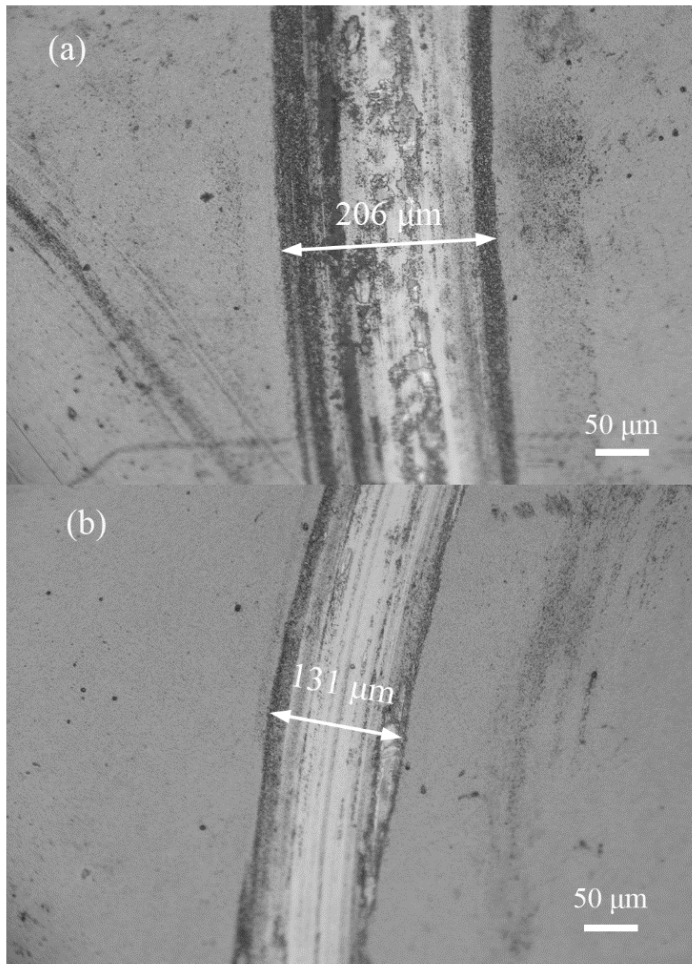

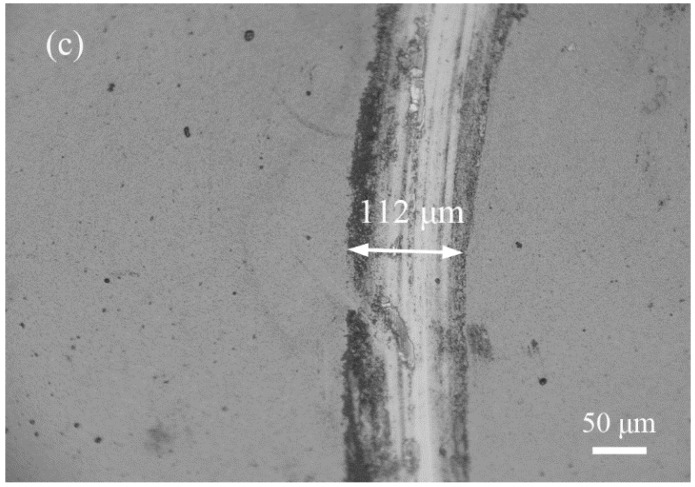
The wear tracks for the films after ball on disc wear tests against SiN ball: (**a**) DCMS (**b**) HP + DC (**c**) HPPMS.

The wear tracks of TiN films by three modes were observed by optical microscopy (OM). Deep and wide grooves with some obvious debris were formed on the film by DCMS mode (Figure 7a), which indicates that abrasive wear occurred accompanied by adhesion wear. Figure 7b presents a relatively light and narrow grooves with tiny debris, suggesting that the wear mechanism is dominated by abrasive wear in HP + DC mode. Meanwhile, the film by HPPMS mode exhibits a smooth surface with the lightest grooves (Figure 7c), identified by abrasive wear. The optical images are in good agreement with the friction coefficient results, which proves that the film deposited by HPPMS mode possesses excellent tribological characteristics.

The HP + DC mode provides high energy ion bombardment during the Ti interlayer growth, which shows a certain improvement in surface roughness, porosity, hardness, and adhesion strength, as compared to DCMS mode. Further, HPPMS-deposited TiN films show superior performance in structure and mechanical performance, therefore resulting in enhanced tribology resistance.

### 3.4. Adhesion

**Figure 8:**
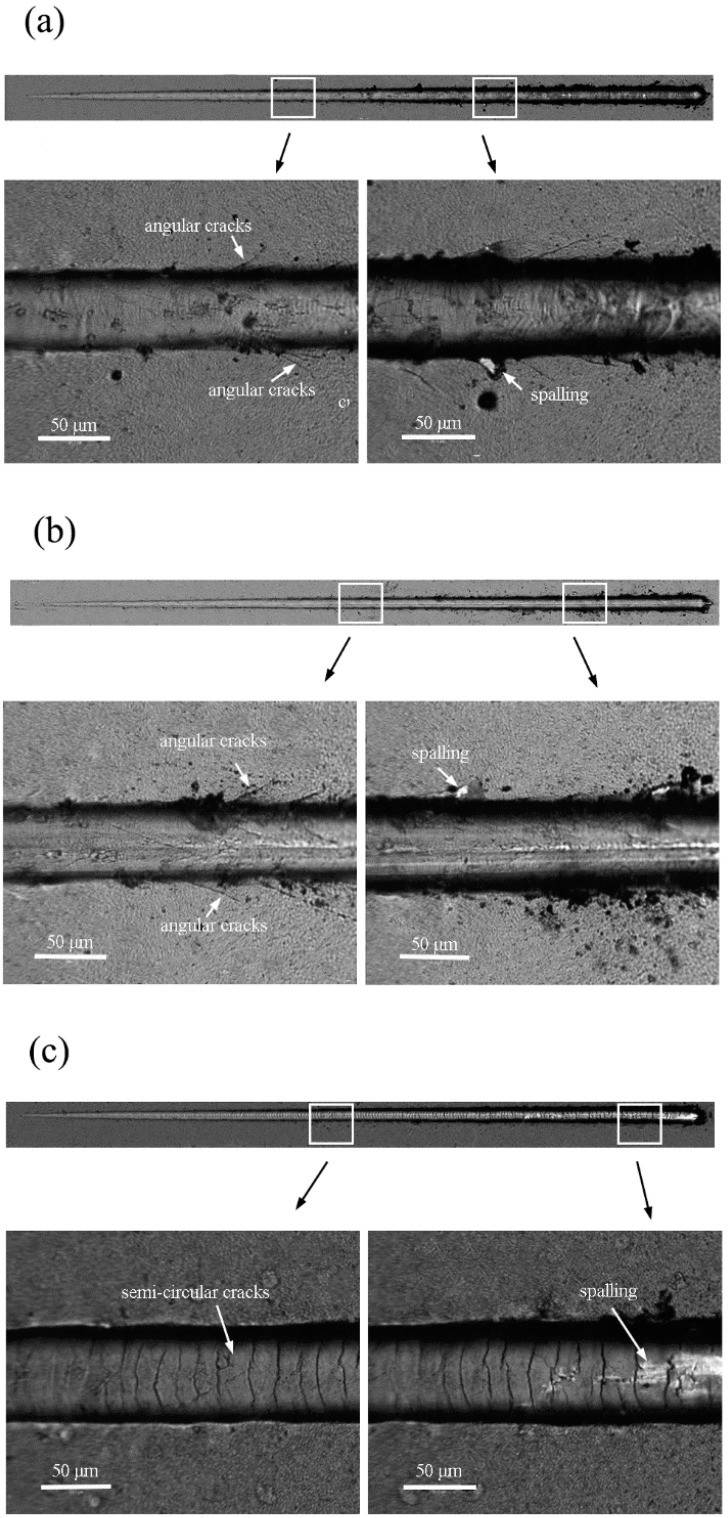
The optical micrographs of scratches TiN film by different modes after scratch test: (**a**) DCMS (**b**) HP + DC (**c**) HPPMS.

**Figure 9:**
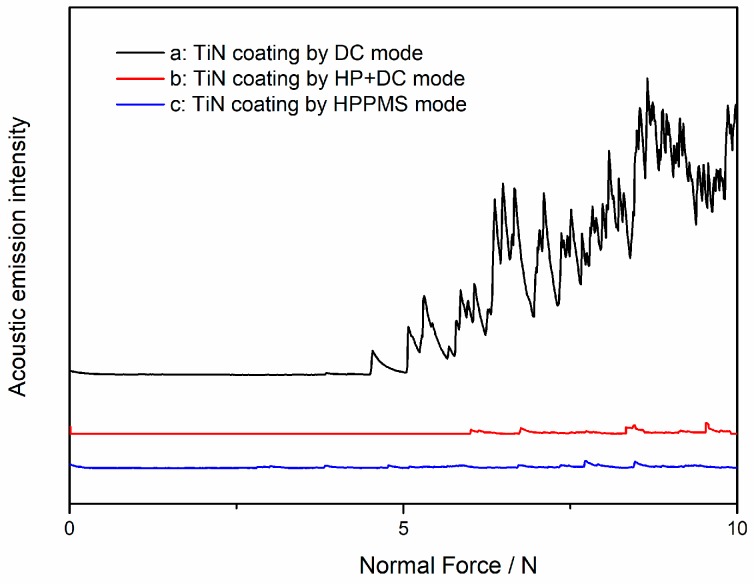
The acoustic emission signals generated by TiN film with different modes during scratch test: (**a**) DCMS (**b**) HP + DC (**c**) HPPMS.

To observe and quantify the adhesion strength of TiN film by different deposition modes, progressive micro-scratch tests were performed under the same condition (applied normal load increased linearly from 0 to 10 N). The optical micrographs of scratches TiN film by different modes after scratch test are shown in Figure 8 and the generated acoustic emission signals during scratch test are shown in Figure 9. It suggests that TiN films that were deposited with mode 1 and 2 experienced similar failure modes [26]. In DCMS TiN, the angular cracks were clearly observed initiating from the edge of scratch at a very low load of 4 N. Then, some new angular cracks were constantly being produced along the edge of scratch as the load progressed. The angle between the angular crack and the forward direction of the diamond scribing head was almost maintained at 45°. At the load of 5.5 N, the signs of chipping appeared at the area between the angular crack and the edge of scratch from film-substrate interface. The results show that the film-substrate interface close to the edge of scratch was damaged with the appearance of angular crack and failed at a critical load of 5.5 N. While, the pre-deposition treatment of Ti interlayer using HP has a positive effect on adhesion strength of the TiN film by improving the critical load to almost 8 N. The TiN film that was produced by HPPMS mode shows completely different failure modes under the same conditions. There were no angular cracks observed in Figure 8c. The semi-circular brittle cracks formed at the rear of the indenter (Figure 8c) in response to the tensile stresses that are generated during sliding, which played as typical failure throughout the remaining process until spalling occurs at the load of 16 N. Obviously, the HP deposition mode improved the scratch resistance of TiN film significantly, which may also attribute to the enhanced ion bombardment during the HPPMS mode, which may make metal ions implanted and incorporated in the substrate, and hence has an enhancement on interface.

### 3.5. Corrosion Resistance

**Figure 10:**
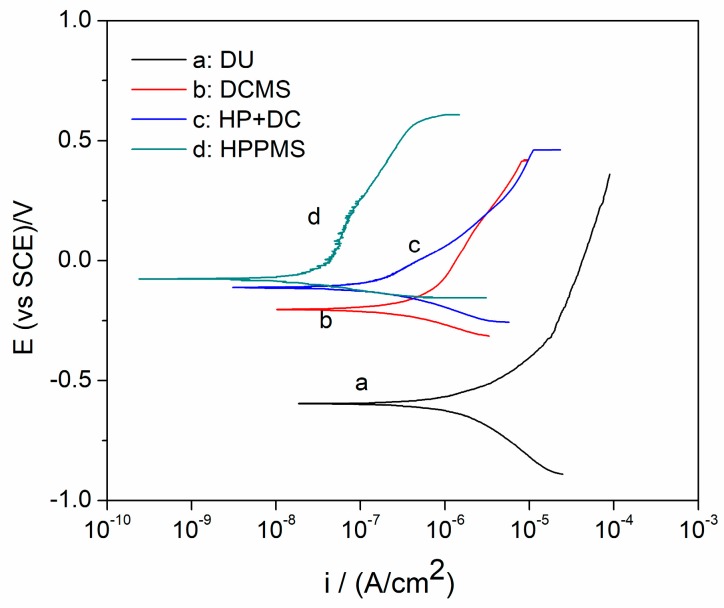
Potentiodynamic polarization curves of DU substrate and TiN films by different modes: (**a**) DCMS, (**b**) HP + DC, (**c**) HPPMS, and (**d**) depleted uranium (DU).

Figure 10 presents the potentiodynamic polarization curves of DU and the DU coated with TiN by different modes in aerated 50 ug/g NaCl solution and Table 5 summarizes the corresponding electrochemical corrosion parameters. A highly active dissolution behavior with regard to the bare DU was observed in curve a. Above the corrosion potential (Ecorr), the anodic current density increased sharply and it finally reached the limiting current density of approximately 3.4 × 10^−6^ A/cm^2^. TiN films could prevent DU from rapid corrosion, as demonstrated by curve b, c, d. Especially, all of the TiN films were characterized by a passive region. However, the current density (icorr) of HPPMS TiN (2.6 × 10^−8^ A/cm^2^) was lower by an order of magnitude than that of HP + DC TiN (2.7 × 10^−7^ A/cm^2^) and two orders of magnitude than that of DCMS TiN (5.1 × 10^−7^ A/cm^2^), respectively. As reported, the electrochemical resistance of film is strongly related to the microstructure and surface morphology [11,27]. The relatively lower corrosion resistance of the DCMS TiN film is mainly due to its porous columnar structure so that the solution can easily reach the DU substrate via the pinholes. The denser structure in HP + DC TiN films contributes to the enhanced electrochemical resistance as compared to DCMS TiN. In addition, HPPMS TiN provides a competitive growth structure with few defects and the lowest porosity, which accounts for the best corrosion resistance.

## 4. Conclusions

In this study, TiN film has been deposited on DU under low temperature by direct current magnetron sputtering (DCMS) and high power pulsed magnetron sputtering (HPPMS), respectively.

The experimental results shown in this work demonstrate that the HPPMS deposited TiN film exhibited a compact and smooth surface as compared to DCMS that was deposited TiN. The DCMS deposited TiN films exhibited porous columnar structure, which corresponds to zone I in the well-known structure zone model (SZM); while, the HPPMS counterpart shows competitive texture growth, which corresponds to zone T in SZM. The preferred orientation of DCMS produced TiN films changed from (200) to (111) with HPPMS. The HPPMS fabricated TiN film reveals a high hardness of 22.09 GPa, Young’s modulus of 220.21, low friction coefficient of 0.34, high adhesion of *L*c 16 N, and an improved corrosion resistance. Besides, the process of Ti interlayer deposition by HPPMS can enhance the film properties as compared to DCMS, which is attributed to the enhanced ion bombardment during the HPPMS.

In summary, the results that are shown in this work prove that the TiN film deposited by HPPMS on DU could significantly improves its mechanical, tribological and corrosion performance and therefore serves as a promising surface protective coating for DU.

## Figures and Tables

**Table 1:** Deposition parameters for TiN film fabricated by direct current magnetron sputtering (DCMS), HP + DC, and high power pulsed magnetron sputtering (HPPMS) modes.

Deposition Mode	Distance to Targets (mm)	Ratio of Ar/N_2_	Ti Inter-Layer		TiN Film		Deposition Rate (nm/min)
			Time (min)	Deposition Mode	Time (min)	Deposition Mode	
1	150	160/18	5	DCMS	35	DCMS	62
2	150	160/18	5	HPPMS	35	DCMS	67
3	150	160/18	5	HPPMS	70	HPPMS	28

**Table 2:** Parameters of the tribological test.

Tribology Pair	Load (g)	Speed (r/min)	Radius of Balls (mm)
Ф6 mm SiN	100	160	4

**Table 3:** Surface roughness (*R*_a_), hardness and Young’s modulus of TiN film by different deposition modes.

Deposition Mode	DCMS	HP + DC	HPPMS
*R*_a_ (nm)	46.65 ± 11.27	37.14 ± 6.65	25.89 ± 5.29
Hardness (GPa)	15.75 ± 0.41	20.56 ± 0.76	22.09 ± 0.39
Modulus (GPa)	163.62 ± 2.21	200.37 ± 4.17	220.21 ± 2.33

**Table 4:** The average tribology coefficient of three deposition modes.

Deposition mode	1	2	3
Average tribology coefficient	0.56	0.42	0.34

**Table 5:** The electrochemical corrosion parameters of DU and DU coated with TiN by different modes: (a) DCMS, (b) HP + DC, and (c) HPPMS in aerated 50 ug/g NaCl solution.

Deposition Mode	Ecorr (mV)	Icorr (A/cm^2^)
DU	−773 ± 97	3.4 ± 0.7 × 10^−6^
DCMS	−230 ± 21	5.1 ± 3.8 × 10^−7^
HP + DC	−113 ± 14	2.7 ± 1.4 × 10^−7^
HPPMS	−87 ± 19	2.6 ± 1.1 × 10^−8^
